# Factors associated with decision-making power of married women to use family planning in sub-Saharan Africa: a multilevel analysis of demographic health surveys

**DOI:** 10.1186/s12889-022-13251-4

**Published:** 2022-04-26

**Authors:** Getu Debalkie Demissie, Yonas Akalu, Abebaw Addis Gelagay, Wallelign Alemnew, Yigizie Yeshaw

**Affiliations:** 1 Department of Health Education and Behavioral Sciences, Institute of Public Health, College of Medicine and Health Sciences, University of Gondar, P. O. Box, 196 Gondar, Ethiopia; 2 Department of Human Physiology, School of Medicine, College of Medicine and Health Sciences, University of Gondar, P. O. Box, 196 Gondar, Ethiopia; 3 Department of Reproductive health, Institute of Public Health, College of Medicine and Health Sciences, University of Gondar, P. O. Box, 196 Gondar, Ethiopia; 4 Department of Epidemiology and Biostatistics, Institute of Public Health, College of Medicine and Health Sciences, University of Gondar, P. O. Box, 196 Gondar, Ethiopia

**Keywords:** Decision-making power, Women, Family planning, Sub-Saharan Africa

## Abstract

**Background:**

In sub-Saharan Africa, there are several socio-economic and cultural factors which affect women’s ability to make decision regarding their own health including the use of contraceptives. Therefore, the main aim of this study was to determine factors associated with decision-making power of married women to use family planning service (contraceptives) in sub-Saharan Africa.

**Methods:**

The appended, most recent demographic and health survey datasets of 35 sub-Saharan countries were used. A total weighted sample of 83,882 women were included in the study. Both bivariable and multivariable multilevel logistic regression were done to determine the associated factors of decision-making power of married women to use family planning service in sub-Saharan countries. The Odds Ratio (OR) with a 95% Confidence Interval (CI) was calculated for those potential variables included in the final model.

**Results:**

Married women with primary education (AOR = 1.24; CI:1.16,1.32), secondary education (AOR = 1.31; CI:1.22,1.41), higher education (AOR = 1.36; CI:1.20,1.53), media exposure (AOR = 1.08; CI: 1.03, 1.13), currently working (AOR = 1.27; CI: 1.20, 1.33), 1–3 antenatal care visits (AOR = 1.12; CI:1.05,1.20), ≥ 4 ANC visits (AOR = 1.14;CI:1.07,1.21), informed about family planning (AOR = 1.09; CI: 1.04, 1.15), having less than 3 children (AOR = 1.12; CI: 1.02, 1.23) and 3–5 children (AOR = 1.08; CI: 1.01, 1.16) had higher odds of decision-making power to use family planning.

Mothers who are 15–19 (AOR = 0.61; CI: 0.52, 0.72), 20–24 (AOR = 0.69; CI: 0.60, 0.79), 25–29 (AOR = 0.74; CI: 0.66, 0.84), and 30–34 years of age (AOR = 0.82; CI: 0.73, 0.92) had reduced odds off decision-making power to use family planning as compared to their counterparts.

**Conclusion:**

Age, women’s level of education, occupation of women and their husbands, wealth index, media exposure, ANC visit, fertility preference, husband’s desire in terms of number of children, region and information about family planning were factors associated with decision-making power to use family planning among married women.

## Background

Sub-Saharan Africa (SSA) accounted for 66% of the maternal deaths globally and had the highest Maternal Mortality Ratio (MMR) at 546 maternal deaths per 100,000 live births [1]. Unplanned pregnancy and short inter-pregnancy spacing are the leading causes of maternal and child death in this region. In developing countries, more than 222 million women’s pregnancies are unplanned [2]. The use of modern family planning methods after delivery is considered an important part of interventional efforts [3, 4].

The 2030 Agenda for Sustainable Development Goal (SDGs) includes relevant targets for using contraceptives under the broader goals of health and well-being of the population and gender equality [5, 6]. Family planning service contributes not only to the reduction of morbidity and mortality of mothers and children, but also prevents the risk of unintended pregnancy and its adverse consequence including HIV/AIDS and abortion and hence, it has been used to improve the standard of living [7]. A data from 51 surveys conducted between 2006 and 2013 showed that although 30% of maternal deaths and 10% of child death could be avoided by extending pregnancy [8], 41% of women in SSA who intended to use modern contraceptives were not using them [9]. Moreover, in 2010 only 17% of married women are using contraceptives in SSA which is too low as compared to North Africa (50%), Middle East (39%), East Asia (76%) and Latin America (68%) [10, 11].

A woman’s ability to choose the method of modern contraceptives is affected by her self-image and sense of empowerment. A woman who feels that she is unable to control other aspects of her life may be less likely to feel that she can make decisions about fertility [12]. Independent or joint decision-making with partners on family planning use has a substantial contribution to the improvement of maternal health [13]. Although women’s empowerment is the key to use contraceptives, unfortunately, women’s position in all aspects of decision-making, including the use of contraceptives, in developing countries is inferior to their husbands or partners [12, 14].

Women often have less decision-making power due to their political, economic, and sociocultural status and may not be in a position to protect themselves from unwanted sexual intercourse and gender-based violence, which may predispose them to sexually transmitted infections and other sexual and reproductive health (SRH) problems [15].

Women decision-making power has a great impact on health care services utilization including family planning service. Studies conducted in rural Nepal [16], Pakistan [17] and Ghana [18] showed that women’s decision-making power plays an important role in determining uptake of maternal health services. One of the reasons for not using contraceptives is they have no power to decide on the use of these service [19]. Evidences showed that women who have decision-making power are more likely to use contraceptives than those who had not [20, 21]. However, decisions for the use of contraceptives may be affected by unbalanced power relations between women and their partners, especially in more male-controlled societies and where cultural discrimination are practiced [22].

Furthermore, previous studies showed that decision-making power of women to use family planning was associated with education [2, 23–25], age [2, 25–28], knowledge about family planning [26, 29, 30], working status of women [27, 28, 31], gender equality attitude [29], number of living children [23, 27, 28], socio-economic status [24, 25, 31–33], residence [27, 28], husbands desire in terms of number of children [30] and attitude towards family planning [26].

Decision-making power of women to use family planning service is a huge problem in SSA region. However, to the best of our knowledge, there is no study that investigates the factors associated with decision-making power to use family planning among married women in the region. Hence, this study was conducted to fill this gap by identifying the determinants of women decision- making power on the use of family planning service in the region. The finding of this study will be helpful to design appropriate intervention measures that can increase the decision -making power of women to use family planning in the region.

## Methods

### Data source

This study used the most recent appended demographic and health survey (DHS) datasets of 35 sub-Saharan countries which were conducted from 2009 to 2018. The DHS is a nationally representative survey, collected every 5 years, to provide population and health indicators at the national and regional levels. A pretested standard demographic and health survey questionnaires were used. The questionnaire was contextualized to the different countries context and the data were gathered by trained data collectors. The datasets of each sub Saharan country were obtained at https://dhsprogram.com/data/dataset_admin/index.cfm. Those countries with no data on decision-making power of women to use family planning were excluded from the analysis. In this study, 83,882 women were included (Table 1).

**Table 1:** List of sub-Saharan countries included, and their demographic and health surveys’ year

Name of Country	Year of survey	Weighted sample size (%)
Angola	2015/16	1083 (1.29)
Burkina Faso	2010	2194 (2.62)
Benin	2017/18	1732 (2.06)
Burundi	2016/17	2792 (3.33)
Cameroon	2018	1497 (1.79)
DR Congo	2013/14	2422 (2.89)
Chad	2015	710 (0.85)
Comoros	2012	631 (0.75)
Congo	2011/12	2813 (3.35)
Côte d’Ivoire	2011/12	1103 (1.32)
Ethiopia	2016	3668 (4.37)
Gabon	2012	1394 (1.66)
Ghana	2014	1415 (1.69)
Gambia	2013	571 (0.68)
Guinea	2018	840 (1.00)
Kenya	2014	5035 (6.00)
Liberia	2013	1090 (1.30)
Lesotho	2014	2168 (2.58)
Madagascar	2009	4807 (5.73)
Mali	2018	1477 (1.76)
Malawi	2015/16	9552 (11.39)
Mozambique	2011	899 (1.08)
Nigeria	2018	4843 (5.77)
Niger	2012	1373 (1.64)
Namibia	2013	1721 (2.05)
Rwanda	2014/15	3706 (4.42)
Sierra Leone	2016	2064 (2.46)
Sao Tome and Principe	2009	607 (0.72)
Senegal	2011	1359 (1.62)
Togo	2013	1231 (1.47)
Tanzania	2015/16	3149 (3.75)
Uganda	2016	4372 (5.21)
South Africa	2016	1663 (1.98)
Zambia	2018	3794 (4.52)
Zimbabwe	2015	4107 (4.90)

### Variables of the study

#### Dependent variable

The dependent variable for this study was decision-making power of married women to use family planning service. According to DHS, decision-making power of married women to use family planning was reported in four categories (decision-making by women, partner, joint and others). Hence, we dichotomized this variable as: yes (if the women decide independently or together with their partner to use family planning) and no (if neither the women decide independently nor jointly with their partner to use family planning) [26].

#### Independent variables

Both individual and community level variables were considered independent variables. The individual level variables were age, level of education, wealth index, occupational status of women and their husbands, media exposure, ANC visit, number of living children, fertility preference of women, husband’s desire in terms of number of children, information related to FP at health facility, residence and SSA region. Countries were categorized in to sub-regions based on socioeconomic and geographical directions [34].

#### Data analysis procedure

We used STATA 14 software to extract, recode and analyze the data. The data were weighted before doing any statistical analysis to restore the representativeness of the sample and to get a reliable estimate and standard error. The whole procedure of weighting and its rationale is found on the guide of DHS statistics [35].

Due to the correlated nature of DHS data, measures of community variation/random-effects such as Median Odds Ratio (MOR), Interclass Correlation Coefficient (ICC), and Proportional Change in Variance (PCV) were calculated. Accordingly, the values of these measures were found out to be significant, and hence the use of multilevel logistic regression model is more appropriate than using ordinary logistic regression. To choose the best fitted model, first we developed four models and compared them with Deviance. These were: the null-model, a model with no independent variable; model I, a model that has individual-level factors only; model II, a model with community-level factors only and model III, a model that contains both community level and independent variables. Model III was selected as the best fitted model as it had the lowest Deviance.

Bivariable and multivariable multilevel logistic regression was performed to determine the associated factors of decision-making power of married women to use FP in SSA. All variables with a *p* value < 0.25 during bi-variable analysis were entered into the multivariable logistic regression model. In the final model, *p* value ≤0.05 was used to declare variables that are statistically significant.

## Results

### Sociodemographic characteristics of the respondents

The total weighted sample of 83,882 married women were included in this study. Of these, 22.9% of the respondents were in the age group of 25–29 years and more than half (60%) of them were rural dwellers. More than one-third of both the respondents (39.9%) and their husbands (34.4%) had primary education. The majority of the respondents (73.3%) and their husbands (92.7%) were currently employed. Similarly, the majority of the respondents (67%) had media exposure (Table 2).

**Table 2:** Sociodemographic characteristics of the respondents in sub-Saharan Africa

Variable	Category	Weighted frequency	Percent (%)
Age (in years)	15–19	3215	3.8
	20–24	13,693	16.0
	25–29	19,210	22.9
	30–34	17,890	21.0
	35–39	15,016	17.9
	40–44	9794	11.9
	45–49	5063	6.5
Residence	Urban	33,704	40.0
	Rural	50,177	60.0
Region	East Africa	37,713	44.7
	West Africa	19,471	24.0
	South Africa	15,430	18.6
	Central Africa	10,534	12.7
Educational level of respondents	No formal education	16,714	19.9
	Primary	33,490	39.9
	Secondary	28,100	33.5
	Higher	5578	6.7
Education level of husbands	No formal education	15,251	18.2
	Primary	28,865	34.4
	Secondary	30,280	36.0
	Higher	9406	11.0
Respondents’ occupation	Currently working	61,454	73.3
	Currently Not working	22,428	26.7
Husbands occupation	Currently working	77,741	92.7
	Currently Not working	6141	7.3
Wealth index	Poorest	10,934	13
	Poorer	14,093	16.8
	Middle	16,119	19.4
	Richer	19,309	23
	Richest	23,425	27.8
Media exposure	Yes	56,269	67.0
	No	27,585	33.0

### Reproductive characteristics of the respondents

Of the respondents, 47.6% of them had four or more ANC visits. The majority of the respondents (69.2%) were told about family planning methods during their facility visits. More than half of the respondents (56%) had fertility preference to have more children. Regarding the use of contraceptive methods, 36.2% of the respondents used injections (Table 3).

**Table 3:** Reproductive characteristics of the respondents in sub-Saharan Africa

Variables	Category	Frequency	Percent (%)
Number of ANC visits	0	24,679	29.4
	1–3	19,255	23
	≥ 4	39,948	47.6
Number of living children	< 3	32,908	39.2
	3–5	38,515	45.9
	> 5	12,458	14.9
Fertility preference	Unable to have children	5100	6
	Do not want another children	31,887	38
	Want to have another children	46,895	56
Information about FP at health facility	Yes	25,844	30.8
	No	58,037	69.2
Husband’s desire in terms of number of children	Same as spouse	37,187	44
	Husband wants more	39,023	46.5
	Husband wants fewer	7671	9.5
Type of contraceptive used	Pill	12,892	15.4
	IUD	2292	2.73
	Injections	30,380	36.2
	Male condom	7236	8.6
	Female sterilization	4305	5
	Periodic abstinence	6703	8
	Withdrawal	3777	4.5
	Implants /Norplant	12,311	14.7
	Lactation amenorrhea (LAM)	1776	2.2
	Other methods (including traditional method)	2205	2.7

### Random effect analysis

The random-effects model result showed that there is significant clustering of decision-making power of women to use family planning across the communities (OR of community level variance =0.07, 95% CI = 0.06–0.10). The value of ICC in the null model revealed that 2.16% of the overall variation of decision-making power of women to use family planning was attributed to cluster variability. The 1.23 MOR value of the null model also indicated the presence of variation in the decision-making power of women to use family planning between clusters. It means if we randomly select women from different clusters, those women at the cluster with higher decision making power of women to use family planning had 1.23 times higher chance of decision-making power to use family planning compared to their counterparts. As you can see in Table 3 below, model III has the lowest Deviance value. Hence, it was selected as the best fitted model (Table 4).

**Table 4:** Comparison of models and result of random effect analysis

Parameters	Null model	Model I	Model II	Model III
Community level variance	0.07 (0.06–0.10)	0.07 (0.06–0.10)	0.07 (0.05–0.09)	0.07 (0.05–0.09)
ICC	2.16%	2.14%	2.00%	2.02%
MOR	1.29	1.29	1.28	1.28
PCV	Ref	0.69%	7.42%	6.60%
**Model fitness**				
Deviance	54,592.124	53,855.498	53,915.178	53,310.886

### Factors associated with decision-making power of women to use contraceptives

The odds of decision-making power to use family planning among married women with age 15–19, 20–24, 25–29 and 30–34 years was decreased by 39% (AOR = 0.61; CI: 0.52, 0.72), 31% (AOR = 0.69; CI: 0.60, 0.79), 26% (AOR = 0.74; CI: 0.66, 0.84), and 18% (AOR = 0.82; CI:0.73, 0.92) as compared to their counterparts, respectively. The odds of decision-making power to use family planning among married women whose education level was primary, secondary and higher was about 1.24 (AOR = 1.24; CI:1.16,1.32), 1.31 (AOR = 1.31; CI:1.22,1.41) and 1.36 (AOR = 1.36; CI:1.20,1.53) times higher compared to those who did not have formal education.

Women who are currently working were 1.27 (AOR = 1.27; CI: 1.20, 1.33) times more likely to have decision-making power to use contraceptive as compared to women who were not currently working. Women who had media exposure were 1.1 (AOR = 1.08; CI: 1.03, 1.13) times more likely to have decision-making power on family planning use as compared to those women who did not have media exposure.

Similarly, the odds of decision-making power on family planning among participants who had 1–3 and ≥ 4 ANC visit was increased by 12%(AOR = 1.12; CI:1.05,1.20) and 14% (AOR = 1.14;CI:1.07,1.21) than those who had no ANC visit, respectively. Besides, the odds of decision-making power on family planning among respondents who were informed about family planning was increased by 9% (AOR = 1.09; CI: 1.04, 1.15) than their counterparts. Women whose husbands desired fewer children had a 14% (AOR = 0.86; CI: 0.79, 0.93) reduced chance of decision- making power for family planning than their counterparts.

Women who had less than 3 and 3–5 children were 1.12 (AOR = 1.12; CI: 1.02, 1.23) and 1.08 (AOR = 1.08; CI: 1.01, 1.16) times higher odds of decision-making power to use family planning than women who had > 5 children, respectively. Women who did not have children had 48% reduced odds of decision-making power to use FP than women who want to have children (AOR = 0.52; CI: 0.47–0.58). Moreover, the odds of decision-making power to use FP was increased by 1.10 (AOR = 1.10; CI: 1.04, 1.17) times among respondents who do not want other children than those who want to have other children (Table 5).

**Table 5:** Multilevel regression analysis of decision-making power to use family planning among married women in sub-Saharan Africa

	Decision-making power		Odds Ratio	
Variables	Yes, No (%)	No, No (%)	COR(95%CI)	AOR(95%CI)
Age (years)				
15–19	2797 (87)	418 (13)	0.76 (0.66–0.87)	0.61 (0.52–0.72)*
20–24	12,167 (88.9)	1526 (11.1)	0.91 (0.81–1.00)	0.69 (0.60–0.79)*
25–29	17,144 (89.3)	2066 (10.7)	0.96 (0.87–1.07)	0.74 (0.66–0.84)*
30–34	16,092 (89.9)	1799 (10.1)	1.04 (0.94–1.05)	0.82 (0.73–0.92)*
35–39	13,506 (89.9)	1510 (10.1)	1.06 (0.95–1.17)	0.89 (0.79–1.00)
40–44	8798 (89.8)	996 (10.2)	1.01 (0.91–1.13)	0.92 (0.82–1.02)
45–49	4527 (89.5)	536 (10.6)	1	1
Residence				
Urban	30,082 (89.3)	3622 (10.7)	1	1
Rural	5229 (10.4)	44,949 (89.6)	1.01 (0.97–1.06)	1.02 (0.96–1.08)
Region				
East Africa	34,861 (92.4)	2853 (7.6)	1	1
West Africa	17,302 (85.6)	2901 (14.4)	0.52 (0.49–0.53)	0.52 (0.49–0.56)*
South Africa	13,895 (90.1)	1536 (9.9)	0.76 (0.71–0.81)	0.76 (0.71–0.82)*
Central Africa	8973 (84.2)	1561 (14.8)	0.51 (0.48–0.55)	0.51 (0.47–0.55)*
Educational level of respondents				
No education	14,423 (86.3)	2291 (13.7)	1	1
Primary	30,266 (90.4)	3224 (9.6)	1.51 (1.42–1.59)	1.24 (1.16–1.32)*
Secondary	25,250 (89.9)	2851 (10.1)	1.40 (1.32–1.49)	1.31 (1.22–1.41)*
Higher	5093 (91.3)	485 (8.7)	1.66 (1.49–1.85)	1.36 (1.20–1.53)*
Respondents’ occupations				
Working	55,489 (90.3)	5965 (9.7)	1.35 (1.29–1.42)	1.27 (1.20–1.33)*
Not working	19,542 (87)	2886 (13)	1	1
Husband’s occupation				
Working	69,705 (89.7)	8035 (10.3)	1.24 (1.15–1.35)	1.17 (1.08–1.27)*
Not working	5326 (86.7)	816 (13.30	1	1
Wealth index				
Poorest	9656 (88.3)	1277 (11.7)	1	1
Poorer	1558 (11.1)	12,535 (88.9)	1.06 (0.98–1.18)	1.01 (0.93–1.09)
Middle	1736 (11)	14,383 (89)	1.09 (1.01–1.18)	1.01 (0.94–1.09)
Richer	2071 (10.3)	17,239 (89.3)	1.12 (1.04–1.20)	1.02 (0.94–1.11)
Richest	2208 (9.4)	21,217 (0.6)	1.28 (1.19–1.38)	1.13 (1.03–1.23)*
Media exposure				
Yes	50,675 (90.1)	5594 (10.9)	1.19 (1.14–1.26)	1.08 (1.03–1.13)*
No	24,335 (88.2)	3251 (11.8)	1	1
ANC visit				
No ANC visit	21,972 (89)	2708 (11)	1	1
1–3 ANC visit	1964 (10.2)	17,291 (89.8)	1.08 (1.02–1.51)	1.12 (1.05–1.20)*
≥ 4 ANC visit	4179 (10.5)	35,769 (89.5)	1.06 (1.01–1.12)	1.14 (1.07–1.21)*
Number of living children				
< 3	29,429 (89)	3749 (11)	1.06 (0.99–1.13)	1.12 (1.02–1.23)*
3–5	34,552 (89.7)	3964 (10.3)	1.09 (1.02–1.17)	1.08 (1.01–1.16)*
> 5	11,050 (88.7)	1408 (11.3)	1	1
Fertility preference				
Who did not have children	4300 (84.3)	800 (15.7)	0.68 (0.63–0.74)	0.52 (0.47–0.58)*
Do not want other children	29,101 (91.3)	2786 (8.7)	1.29 (1.23–1.36)	1.10 (1.04–1.17)*
Want to have other children	41,630 (88,8)	5265 (11.2)	1	1
Women who are told FP at health facility				
Yes	23,291 (90.1)	2553 (9.9)	1.13 (1.08–1.19)	1.09 (1.04–1.15)*
No	51,739 (89.2)	6298 (10.8)	1	1
Husbands’ desire in terms of number of children				
The same with spouse	33,656 (90.5)	3531 (9.5)	1.22 (1.17–1.28)	0.99 (0.94–1.04)
Husbands who wants more	34,518 (88.5)	4505 (11.5)	1	1
Husbands who wants fewer	6856 (89.4)	815 (10.6)	1.07 (0.98–1.16)	0.86 (0.79–0.93)*

*P-value≤0.05

## Discussion

The main aim of this study was to determine associated factors of decision-making power to use family planning among married women in sub-Saharan Africa. Accordingly, in this study age, level of education of women, women and their husbands’ occupation, wealth index, region, media exposure, ANC visit, fertility preference of women, husbands’ desire in terms of the number of children and information about family planning were factors associated with decision-making power of women to use family planning.

As this study showed, older women were more likely to decide to use family planning service than the younger ones. This finding is similar to a study conducted in Ethiopia [28], Mozambique [19] and Bangladesh [36]. A possible explanation is that when women get older, they may feel more confident to deal with their husband and to decide on family planning use [37]. On the other hand, young women might not be expected to argue with their older husbands and are required to respect their opinions which may lead to the low decision-making power of younger women to use FP.

The present study revealed that educational status of women was associated with decision- making power of women to use FP. Consistently, other studies also showed that educated women had higher odds of decision-making power to use family planning [2, 27, 37, 38]. Education improves women’s control over their reproductive choices by increasing their position within the family and educated women are more likely to desire smaller families than others and hence have a stronger motivation to practice contraceptives [39].

This study also showed that those women and their husbands who were currently working contribute to decision-making power of women to use FP. This finding is similar with other studies in Malawi [40], Ethiopia [2], Nigeria [41] and South Asia [42]. Women who have occupations may have power and resources, consequently leading to increased independence. Therefore, they do not have to depend on their spouses for resources to make decisions and buy contraceptives. Besides, women whose husbands had occupation may improve the family life generally and this may contribute to women’s decision-making power to use FP indirectly.

Similarly wealth index was positively associated with decision-making power of women. Those women from the richest wealth index had higher chance of decision-making power to use FP than the poorest ones. This finding is in line with other previous studies which explain that women’s economic status impacts their health and decision-making power on contraceptive usage [32, 43, 44]. Women who had more income may have had access and exposure to mass media about contraceptives and hence it increases the likelihood of women’s decision-making power to use it. Furthermore, in this study, media exposure was associated with women’s decision-making power to use FP which is in line with other previous studies [28, 41]. This is due to the fact that mass media helps to increase the decision-making power of women to use contraceptives [29].

In the present study we observed that women who had more children were less likely to have decision-making power on the use of contraceptives as compared to those who had fewer children. This finding seems odd and in contrast with other studies [23, 27]. This might be related to some religions which teach their followers not to use any modern family planning methods. On the other hand, in this study we also found out those women whose husbands had higher desire for more number of children had poor decision-making power to use FP. This finding was similar to a study conducted in Honduras [45] and Ethiopia [30]. This could be related to husbands’ strong influence on women not to use FP, particularly in developing countries [46, 47].

In this study, women who were informed about FP at a health facility had more decision-making power to use FP as compared to their counterparts. This finding is consistent with other studies [46, 48]. The implication of this finding is those women who have information and knowledge about family planning could help them to discuss about the use of contraceptives and influence their husbands. Similarly this study showed that those women who attended ANC visits were more likely to have decision-making power to use family planning. This finding was also consistent with other studies [24, 36]. One explanation is that women go to health facilities for ANC services where they are receiving health information including family planning.

One strength of this study is the use of a representative dataset that includes 35 sub-Saharan countries, making the findings of this study generalizable to the region. The other strength of the study is the use of multilevel modeling, a model that accounts for the nested/hierarchical nature of the demographic and health survey to get reliable estimates. However, the study has also limitations. Because of the secondary nature of the study, there were some ambiguous measurement of variables in the data that we could not correct at this level which remains as amorphous and we can also only determine associations; no causality as it is an observational study. The other limitation of this study is because of we used DHS conducted in different years, it is impossible to accurately compare results.

## Conclusions

Age, women’s level of education, women and their husbands’ occupation, wealth index, media exposure, ANC visit, fertility preference, husband’s desire for more number of children, region and information about family planning were factors associated with decision-making power to use family planning among married women. Behavior change interventions including health education and promotion in this region should target young married women, women who are not educated, women who are not currently working and whose husbands’ desire to have is more number of children thereby to improve the decision-making power of women to use family planning.

## Data Availability

All the data related to the study were included in the manuscript. The DHS datasets analyzed for this study are available in the DHS repository with its website upon reasonable request. (https://dhsprogram.com/data/dataset_admin/index.cfm).

## Abbreviations

ANC: Antenatal Care
DHS: Demographic and Health Surveys
FP: Family Planning
MMR: Maternal Mortality Ratio
SRH: Sexual Reproductive Health
SSA: Sub-Saharan Africa
WHO: World Health Organization
